# The Role of Age in Shaping Cognitive, Physical, and Psychosocial Outcomes in Hemodialysis Patients: A Cross-Sectional Study

**DOI:** 10.3390/medicina61071295

**Published:** 2025-07-18

**Authors:** Leszek Sułkowski, Andrzej Matyja, Maciej Matyja

**Affiliations:** 1 Department of General Surgery, Regional Specialist Hospital, 42-218 Częstochowa, Poland; leszeksulkowski@szpitalparkitka.com.pl; 2 2nd Department of General Surgery, Jagiellonian University Medical College, 30-688 Kraków, Poland

**Keywords:** aging, chronic kidney disease (CKD), cognitive impairment, cross-sectional study, fatigue, hemodialysis, multidimensional health evaluation, pain assessment, psychosocial factors, quality of life

## Abstract

*Background and Objectives:* Chronic kidney disease frequently progresses to end-stage renal disease, requiring dialysis, which imposes significant physical, psychological, and social burdens. Age is a key factor influencing symptom experience and quality of life in dialysis patients, yet findings on its impact remain mixed. This study aimed to examine how age relates to a broad range of health domains—including fatigue, pain, cognition, mental health, sexual satisfaction, bowel control, visual impairment, social support, and quality of life—among hemodialysis patients. *Materials and Methods:* A cross-sectional study was conducted at a single dialysis center in Poland, involving 79 adult patients undergoing maintenance hemodialysis. Standardized, validated psychometric instruments were used, including the Modified Fatigue Impact Scale (MFIS), Perceived Deficits Questionnaire (PDQ), Pain Effects Scale (PES), Mental Health Inventory (MHI), Modified Social Support Survey (MSSS), Sexual Satisfaction Scale (SSS), Bowel Control Scale (BWCS), Impact of Visual Impairment Scale (IVIS), and WHOQOL-BREF for quality of life. Spearman’s or Pearson’s correlation coefficients were used to evaluate relationships between age and scale scores. Full and abbreviated versions of scales were also compared. *Results:* Age showed moderate positive correlations with fatigue (ρ = 0.44–0.53), cognitive deficits (ρ = 0.37–0.45), pain (r = 0.41), bowel control issues (ρ = 0.32), and visual impairment (ρ = 0.37), all statistically significant (*p* < 0.01). No significant associations were observed between age and mental health (MHI), perceived social support (MSSS), or quality of life (WHOQOL-BREF). Abbreviated versions of the scales showed strong correlations with their full versions (ρ > 0.9). *Conclusions:* While age is linked to increased symptom burden in select domains such as cognition, fatigue, and pain, it does not significantly affect mental health, perceived social support, or overall quality of life in hemodialysis patients. These findings support the use of age-sensitive, multidimensional assessments to inform individualized care strategies.

## 1. Introduction

Chronic kidney disease is a progressive condition that affects millions of individuals worldwide and frequently progresses to end-stage renal disease, which necessitates renal replacement therapy such as hemodialysis or peritoneal dialysis. While dialysis is life-sustaining, it imposes substantial physiological, psychological, and social burdens on patients, significantly impacting their quality of life [1,2]. A growing body of evidence indicates that dialysis patients experience diminished quality of life compared to the general population due to the interplay of comorbid conditions, treatment-related complications, and psychosocial distress [3,4]. Patients undergoing dialysis often report high levels of fatigue, pain, cognitive decline, visual impairment, depression, anxiety, and reduced social support, all of which interfere with daily functioning and long-term health outcomes [1,5,6,7].

Among the many variables influencing dialysis outcomes, age plays a particularly critical role. It affects not only the medical profile of patients but also shapes their psychological resilience, cognitive function, and social relationships [3]. Studies have shown that younger dialysis patients may experience heightened psychological distress as a result of disrupted life plans, employment challenges, and social role changes, while older patients are more susceptible to cognitive decline, frailty, and social isolation [5,6,7]. Age-related differences in coping strategies and emotional regulation further contribute to variability in mental health outcomes [8,9]. In addition to age, other sociodemographic factors such as gender, education, income, and marital status are known to influence health perceptions and treatment outcomes in dialysis populations and may interact with age in complex ways [1,8,9].

Mental health disorders, particularly depression and anxiety, are highly prevalent in the dialysis population and are associated with poor adherence to treatment and reduced quality of life [4,5,6]. In this context, the assessment of mental well-being provides crucial insight into the emotional dimensions of dialysis care.

Cognitive impairment is another major concern, with over half of long-term hemodialysis patients exhibiting some degree of cognitive dysfunction [9,10]. This may be due to cumulative exposure to uremic toxins, hemodynamic instability during dialysis sessions, and age-related neurodegenerative processes [11,12,13,14]. Cognitive symptoms, if unaddressed, can further impair treatment management and reduce functional independence [12,13,15].

Fatigue is one of the most pervasive symptoms reported by dialysis patients and has been shown to compromise both physical activity and psychosocial functioning [15,16]. Although some studies link increasing fatigue severity with older age due to physiological decline and comorbidities, other research finds no significant age-related trend, suggesting the influence of factors such as psychological health and employment status [14,17].

Pain, often underreported in dialysis settings, can severely impact mood, sleep, and functional status. While older patients may report more pain due to musculoskeletal degeneration, younger patients often perceive pain more intensely because of differing expectations and coping abilities [18,19,20].

Visual impairment, though less frequently discussed, is also a prevalent comorbidity in dialysis patients. It reduces autonomy and contributes to overall functional decline, especially in the elderly [13,19,20]. This aspect of patient health is often neglected despite its substantial impact on daily life and mental health.

Social support is a vital determinant of emotional and psychological stability in individuals undergoing dialysis. Older adults often benefit from more consistent support networks, while younger patients may face isolation due to career disruption and changes in peer relationships [21,22].

Quality of life is a multifaceted construct that integrates physical, psychological, social, and environmental domains. Existing literature reports mixed findings on age-related differences: younger patients may report lower quality of life due to greater emotional distress, whereas older individuals often struggle with greater physical limitations and dependency [23,24,25].

Given the complex, multidimensional nature of health challenges in dialysis patients and the potential moderating role of age, this study aims to investigate the association between age and fatigue, pain, sexual satisfaction, bowel control, visual impairment, cognitive function, mental health, social support, and quality of life.

Through the application of validated psychometric tools and robust statistical methodologies, this study seeks to clarify how age influences the physical, cognitive, and emotional dimensions of health in the dialysis population. Understanding these relationships is essential for informing individualized, age-sensitive clinical care strategies and guiding policies that prioritize symptom-specific and patient-centered support across the lifespan.

## 2. Materials and Methods

This study received ethical approval from the Bioethics Committee (approval number: K.B.Cz. 0014/2017). Participants were enrolled from the Dialysis Unit at the Regional Specialist Hospital in Częstochowa, Poland. All individuals were fully informed about the purpose and procedures of the study, and written informed consent was obtained prior to participation. The study was conducted between November and December 2019.

Eligibility criteria included: a diagnosis of end-stage renal disease managed with hemodialysis, age over 18 years, willingness to participate, and provision of signed informed consent. Patients were excluded if they had acute kidney injury, were undergoing peritoneal dialysis, were under the age of 18, declined to participate, or submitted incomplete questionnaire data.

The questionnaires were completed by patients independently, without the presence of others, although a trained interviewer was available on-site to address any questions. Participants were instructed to answer based on their experiences from the preceding four weeks. All questionnaires were administered during the mid-week dialysis session (Wednesday or Thursday) to ensure consistency of patient condition and reduce variability due to intradialytic timing effects.

This study examined the relationship between age and multiple psychological, cognitive, social, and quality-of-life measures in individuals undergoing hemodialysis. A cross-sectional design was employed, and standardized psychometric instruments were used to assess various aspects of patients’ well-being. The analyses included measures of fatigue, pain, sexual satisfaction, bowel control, visual impairment, cognitive function, mental health, social support, and quality of life.

The Modified Fatigue Impact Scale (MFIS) is a validated tool designed to assess the impact of fatigue on daily activities, particularly in patients with chronic conditions. It includes 21 items that evaluate fatigue across three domains: physical, cognitive, and psychosocial functioning [26]. The scale has been widely used in both clinical and research settings due to its strong reliability and validity. A shortened five-item version (MFIS-5) is available for quick screening, but the full version was preferred in this study for its comprehensive assessment of fatigue [27,28].

The Pain Effects Scale (PES) was derived from the Medical Outcomes Study and consists of six items assessing the extent to which pain and unpleasant sensations interfere with mood, mobility, sleep, work, recreational activities, and overall enjoyment of life [27,29]. Previous studies have confirmed its effectiveness in detecting pain-related distress and its impact on mental health in chronic illness populations [30].

The Sexual Satisfaction Scale (SSS) is a four-item scale that evaluates satisfaction with physically expressed affection, variety of sexual activities, and the overall sexual relationship. An additional item assesses perceived partner satisfaction. It was adapted from the Sexual History Form and has demonstrated utility in evaluating sexual adjustment in patients with chronic illnesses, including those on hemodialysis [27,31].

The Bowel Control Scale (BWCS) consists of five items designed to assess the frequency and impact of bowel dysfunction on daily life. It was adapted from the Bowel-Bladder Function Scale and the Sickness Impact Profile, both of which have been validated for use in patients with chronic conditions affecting gastrointestinal function [27,32].

The Impact of Visual Impairment Scale (IVIS) is a five-item instrument designed to measure difficulties related to visual recognition tasks such as reading and performing daily activities, which cannot be corrected with standard visual aids. The scale was adapted from the Functional Capacities Assessment used in ophthalmologic and neurological studies [13,27].

The Perceived Deficits Questionnaire (PDQ) is a 20-item self-report measure designed to assess cognitive function, including attention, retrospective memory, prospective memory, and planning/organization. The scale has been widely used to evaluate cognitive impairment in individuals with neurological conditions, including those undergoing hemodialysis [27,33]. A five-item short version (PDQ-5) is also available and has demonstrated similar psychometric properties [27,34].

The Mental Health Inventory (MHI), originally developed in the National Health Insurance Study, is a validated instrument used to assess mental health status, psychological distress, and emotional well-being [27,35]. The 18-item version was used in this study due to its strong correlation with full-scale mental health assessments while maintaining efficiency in data collection [36].

The Modified Social Support Survey (MSSS) was adapted from the MOS Social Support Survey and measures four domains of social support: emotional/informational support, tangible support, positive social interaction, and affectionate support [27,37]. The full 18-item version was used in this study to ensure a detailed assessment of social support structures in hemodialysis patients.

The WHOQOL-BREF is a validated tool developed by the World Health Organization to assess quality of life across cultures. It includes 26 items grouped into four domains: Physical Health, Psychological Health, Social Relationships, and Environment. Each domain score is calculated as the mean of its items, with higher scores reflecting better quality of life. The instrument addresses various life aspects—physical functioning, emotional well-being, social interactions, and environmental conditions such as safety, resources, and access to services. It can be self-completed or interviewer-assisted, depending on the respondent’s reading ability. Due to its concise format and cross-cultural relevance, the WHOQOL-BREF is widely used in clinical, public health, and policy-related research [38].

### Statistical Analysis

The statistical analysis in this study aimed to examine the relationships between age and various psychometric scales, as well as the correlation between full and abbreviated versions of selected scales. Given the nature of the data and the presence of non-normal distributions and potential nonlinear relationships, non-parametric and parametric statistical methods were chosen accordingly [39,40]. Spearman’s rank correlation coefficient was used to assess monotonic relationships between age and the scores of multiple scales, including their subscales. This method was selected due to its robustness in handling ordinal data, its ability to evaluate relationships without assuming normality or linearity, and its suitability for datasets containing potential outliers. The correlation coefficient (ρ) was computed for each pair of variables, with values ranging from −1 to 1. A positive ρ value indicated a direct relationship, while a negative ρ value suggested an inverse relationship. The strength of the correlation was classified as negligible (<0.1), weak (0.1–0.3), moderate (0.3–0.5), or strong (>0.5). Statistical significance was determined using *p*-values, with a threshold of *p* < 0.05 considered indicative of a significant correlation. In cases where the relationship between age and psychometric scales was expected to follow a linear pattern, Pearson’s correlation coefficient was employed. This method measured the strength and direction of the linear relationship between two continuous variables. The correlation coefficient (r) ranged from −1 to 1, with values closer to −1 or 1 indicating a stronger negative or positive linear relationship, respectively. Normality of the data was assessed using visual inspection and the Shapiro–Wilk test to ensure the appropriateness of this method. As with Spearman’s correlation, statistical significance was determined using *p*-values, with *p* < 0.05 set as the threshold for significance.

To evaluate the relationship between the full and abbreviated versions of certain scales, such as MHI and MHI-5, PDQ and PDQ-5, MSSS and MSSS-5, and MFIS and MFIS-5, Spearman’s correlation coefficient was applied. This approach was used to assess the extent to which the shorter versions reflected the overall variability captured by the full scales. A high correlation coefficient (ρ > 0.9) was considered indicative of a strong agreement between the two versions, supporting the reliability of the abbreviated scales as efficient proxies for their full counterparts. The data analysis was performed using IBM SPSS Statistics (Version 27.0, IBM Corp., Armonk, NY, USA).

## 3. Results

Of the 135 dialysis patients evaluated, 79 (58.5%) met inclusion criteria and therefore were enrolled in the study. The study group included 47 men (59.5%) and 32 women (40.5%), with a mean age of 63.1 years (range: 28–84) (Table 1). Most participants were married (69.6%), while the remaining 30.4% were single. Educational attainment was primarily at the primary level (57.0%), followed by secondary (26.6%) and university education (16.5%).

The majority (87.3%) received hemodialysis through an arteriovenous fistula, with the remainder (12.7%) using a central venous catheter. The average duration of dialysis was 48.1 months, ranging from 1 to 317 months. Hypertension was the most common comorbidity, present in 67.1% of patients, followed by diabetes in 27.8%. A history of kidney transplantation was noted in 7.6% of cases. Key dialysis metrics included an average Kt/V of 1.27 (0.34–2.50), a mean ultrafiltration volume of 2168 mL (400–4000 mL), and a mean urea reduction ratio of 0.65 (0.22–0.89).

### 3.1. Relationship Between Age and Fatigue

A moderate positive correlation was identified between age and all MFIS subscales (Physical, Cognitive, and Psychosocial), as well as the full MFIS and its abbreviated version, MFIS-5 (Table 2). The ρ values ranged from 0.440 to 0.531, all of which were statistically significant (*p* < 0.001). The correlation between the full MFIS and MFIS-5 was particularly strong (ρ = 0.950, *p* < 0.001), confirming the suitability of the shortened scale.

### 3.2. Relationship Between Age and Pain

A moderate positive correlation was observed between age and PES scores, indicating that older patients report greater pain intensity (Table 3).

### 3.3. Relationship Between Age and Sexual Satisfaction

A weak but statistically significant positive correlation was found between age and SSS scores, suggesting that sexual satisfaction slightly increases with age (Table 4).

### 3.4. Relationship Between Age and Bowel Control

A weak-to-moderate positive correlation was found between age and BWCS scores, suggesting a slight increase in bowel control issues with age (Table 5).

### 3.5. Relationship Between Age and Visual Impairment

A weak-to-moderate positive correlation was found between age and IVIS scores, indicating increased visual impairment with age (Table 6).

### 3.6. Relationship Between Age and Cognition

In contrast to the MHI results, a statistically significant association was found between age and the PDQ subscales (Table 7). Moderate positive correlations were observed for all PDQ subscales, with ρ values ranging from 0.374 to 0.445 (*p* < 0.001), suggesting that older individuals reported higher perceived deficits in attention, retrospective memory, prospective memory, and planning. The strong correlation between the full PDQ scale and the abbreviated PDQ-5 (ρ = 0.935, *p* < 0.001) confirmed the reliability of the PDQ-5 in capturing trends observed in the full PDQ scale.

### 3.7. Relationship Between Age and Mental Health

The analysis of the relationship between age and the MHI scores revealed no statistically significant associations. Spearman’s rank correlation coefficient was applied to assess the monotonic relationships between age and the full MHI scale, its subscales (Anxiety, Depression, Behavior Control, and Positive Affect), and the abbreviated version, MHI-5 (Table 8). The results indicated weak positive correlations for all scales, with ρ values ranging from 0.098 to 0.135 and corresponding *p*-values exceeding the significance threshold of 0.05. These findings suggest that age does not substantially influence the MHI scores. In contrast, the correlation between the full MHI scale and its abbreviated counterpart, MHI-5, was found to be very strong (ρ = 0.974, *p* < 0.001), confirming that the MHI-5 serves as a reliable proxy for the full scale.

### 3.8. Relationship Between Age and Social Support

No significant correlations were found between age and the MSSS subscales, including tangible support, emotional support, affectionate support, and positive social interactions (Table 9). The observed correlations were weak and negative, with ρ values between −0.052 and −0.086, none of which reached statistical significance (*p* > 0.05). However, the MSSS-5 showed a very strong correlation with the full MSSS (ρ = 0.992, *p* < 0.001), supporting the use of the abbreviated version as an efficient alternative for assessing social support.

### 3.9. Relationship Between Age and Quality of Life

No significant associations were found between age and the WHOQOL-BREF subscales (Physical, Psychological, Social Relationships, and Environment) (Table 10). The correlation values were weak, ranging from −0.103 to 0.037, and none reached statistical significance (*p* > 0.05). These findings suggest that age does not strongly influence perceived quality of life as measured by this scale.

Overall, the findings suggest that age is moderately associated with increased perceived deficits in cognitive function and fatigue, as indicated by significant correlations in the PDQ and MFIS scales. However, age was not a significant predictor of mental health (MHI), social support (MSSS), or quality of life (WHOQOL-BREF) scores. The strong correlations between the full and abbreviated versions of multiple scales highlight the utility of these shorter instruments for efficient assessment. These results underscore the importance of considering age when interpreting cognitive and fatigue-related measures while also demonstrating the relative stability of mental health, social support, and quality of life across age groups.

## 4. Discussion

This study examined the associations between age and a wide range of psychological, cognitive, physical, and social parameters in hemodialysis patients. The results illustrate a complex and multifactorial relationship, where age significantly affects some aspects of patients’ well-being, while others remain unaffected.

Moderate positive correlations (ρ = 0.440–0.531, *p* < 0.001) were found between age and all MFIS subscales, suggesting that fatigue severity increases with age. This is in line with studies demonstrating that older patients often experience more intense fatigue due to decreased physical resilience and a higher burden of comorbidities [41,42]. Nevertheless, the literature is not uniform; some studies have found no significant correlation, possibly due to the mediating effects of depression, anemia, or dialysis duration [43,44]. The strong correlation between MFIS and MFIS-5 (ρ = 0.950, *p* < 0.001) further supports the validity of the short-form fatigue scale in clinical practice.

A moderate positive correlation (r = 0.413, *p* < 0.001) was observed between age and pain scores, indicating that older patients tend to report more severe pain. This finding is consistent with earlier research highlighting the increased prevalence of pain in older hemodialysis patients due to musculoskeletal issues, neuropathy, and longer exposure to dialysis-related complications [45,46]. However, some studies do not find a direct association between age and pain, emphasizing the importance of individualized assessment and management strategies [46].

Interestingly, a weak but statistically significant positive correlation was found between age and sexual satisfaction (ρ = 0.284, *p* = 0.017). This contradicts much of the literature, which links aging with decreased sexual function due to hormonal and vascular changes [47,48]. However, some studies report that older adults may experience greater sexual satisfaction despite reduced activity, possibly due to evolving expectations, improved communication with partners, or increased emotional intimacy [49].

A weak-to-moderate positive correlation (ρ = 0.315, *p* = 0.008) suggests that bowel control difficulties slightly increase with age. While age-related physiological changes may contribute to this, studies have indicated that other variables—including gender, serum phosphorus, and colonic transit time—may play a more significant role than age alone [50,51].

A weak-to-moderate positive correlation (ρ = 0.370, *p* = 0.002) between age and visual impairment was observed, indicating that older patients experience more pronounced vision problems. This is consistent with the known association between aging and ocular pathologies such as diabetic retinopathy and age-related macular degeneration [13,52]. Given the functional consequences of vision loss in daily living and self-care, these findings highlight the need for routine ophthalmologic evaluations in the dialysis population.

Moderate positive correlations (ρ = 0.374–0.445, *p* < 0.001) were observed between age and all PDQ subscales, suggesting that older individuals report greater cognitive difficulties. This is consistent with prior research indicating that cognitive impairment affects over half of patients undergoing long-term dialysis [16]. These impairments may stem from age-related neurodegeneration, hemodialysis-induced cerebral hypoperfusion, and accumulation of uremic toxins [18,19]. Prolonged dialysis duration has also been associated with worsening memory and executive function, especially in elderly patients [20]. The strong correlation between the full PDQ and the abbreviated PDQ-5 (ρ = 0.935, *p* < 0.001) supports the clinical utility of the shorter form.

No significant correlation was found between age and MHI scores, either on the full scale or its subscales (ρ = 0.098–0.135, *p* > 0.05), indicating that age is not a strong predictor of mental health outcomes in this population. These results are consistent with previous findings showing no clear age-related patterns in mental health among hemodialysis patients [5,13]. Although some studies suggest that younger patients may experience greater psychological distress due to disrupted life goals and social roles [2], others indicate that older adults are more susceptible to depression and anxiety due to comorbidities and social isolation [5,53]. Our findings suggest that other factors—such as treatment adherence, coping mechanisms, and perceived social support—may play a more decisive role than age alone in shaping mental health outcomes in dialysis patients [6,8,13].

We found no significant correlation between age and MSSS scores (ρ = −0.052 to −0.086, *p* > 0.05), indicating that perceived social support does not vary meaningfully across age groups. These findings are consistent with previous studies reporting age-invariant levels of perceived support in dialysis populations [25]. However, the nature of support may shift with age: older adults may rely more on structured, long-standing support systems, while younger patients may be more vulnerable to social disruption and loneliness due to employment loss or altered social roles [54,55]. Importantly, perceived social support has been shown to influence both treatment adherence and survival outcomes, regardless of age [56]. The very high correlation between MSSS and MSSS-5 (ρ = 0.992, *p* < 0.001) validates the use of the short version for practical assessments.

Weak and non-significant correlations (ρ = −0.103 to 0.037, *p* > 0.05) were noted between age and the WHOQOL-BREF subscales. These findings support prior studies indicating inconsistent associations between age and quality of life in dialysis patients [2,25]. While older individuals may experience greater physical limitations, they may also develop stronger coping strategies and adjusted expectations, leading to comparable levels of life satisfaction across age groups [53].

Our study highlights the multifaceted and nuanced relationships between age and various dimensions of well-being in hemodialysis patients. While age was significantly associated with cognitive function, fatigue, pain, bowel control, and visual impairment, it did not appear to substantially influence mental health, perceived social support, overall quality of life, or sexual satisfaction. These findings suggest that age-specific clinical practices may be beneficial. In particular, older hemodialysis patients could benefit from routine cognitive screening, for example, using brief instruments such as the PDQ-5, to detect early signs of cognitive decline that may impact treatment adherence and independence. Similarly, regular ophthalmologic evaluations should be considered to identify and manage vision impairment, which can contribute to falls, medication errors, and reduced quality of life. The strong correlations between full and abbreviated scales also support the integration of short-form screening tools into routine care, allowing for efficient yet comprehensive assessments in busy dialysis units. Future research should explore multivariable, longitudinal models to guide individualized interventions based not only on age but also on comorbidity profiles, treatment history, and psychosocial context.

## 5. Limitations and Perspectives for Future Research

This study provides valuable insights into the impact of age on multiple psychological, cognitive, social, and quality-of-life aspects among patients undergoing hemodialysis. However, several limitations must be acknowledged. One of the primary limitations of this study is its single-center design, which may restrict the generalizability of the findings. While the selected population allowed for a comprehensive assessment, a multicenter study would help confirm the observed relationships across diverse patient populations with varying healthcare settings and social support structures. Future research should aim to replicate these findings in larger, more heterogeneous cohorts to enhance external validity.

Another important limitation is that several potential confounding variables—such as comorbidities, sex, dialysis duration, educational level, income, and dialysis vintage—were not included in the statistical analysis. Although these factors may influence physical, cognitive, and emotional outcomes in hemodialysis patients, the current study focused exclusively on the unadjusted associations between age and multiple health domains. As such, the influence of age could not be examined independently of these variables. While incorporating these confounders into multivariable regression models could yield a more precise understanding of age-related effects, our relatively small sample size limited the feasibility of such analyses without compromising statistical validity through overfitting. Therefore, we opted for bivariate correlation analyses as an exploratory, hypothesis-generating approach to identify general trends. Future studies with larger, more diverse samples should employ multivariable models to account for these confounders and clarify the specific contribution of age.

In addition, no correction for multiple was applied. Given the exploratory nature of the study and the limited sample size, we prioritized sensitivity to detect potential associations across a broad range of domains. However, this approach increases the likelihood of Type I error, particularly for results near the significance threshold. Consequently, these findings should be interpreted with caution and considered hypothesis-generating. Future research with larger samples and prespecified hypotheses should incorporate appropriate correction methods to improve statistical robustness.

Another limitation is the cross-sectional nature of the study. Although our findings highlight important associations between age and various well-being measures, causal relationships cannot be established. Longitudinal studies are needed to explore how these factors evolve over time and whether age-related changes in mental health, cognition, fatigue, and social support dynamically influence treatment outcomes and overall quality of life.

Despite these limitations, a key strength of this study is its holistic approach, evaluating the influence of age on multiple facets of hemodialysis patients’ lives. Unlike previous research, which often focused on single aspects such as depression or cognitive function, our study provides a comprehensive perspective by integrating fatigue, pain, sexual satisfaction, bowel control, visual impairment, cognitive function, mental health, social support, and quality-of-life assessments. This multidimensional approach enhances our understanding of how aging impacts different aspects of well-being in this vulnerable population.

## 6. Summary of Findings

This study investigated the relationship between age and a range of psychological, cognitive, physical, and social parameters in individuals undergoing hemodialysis. The findings highlight the complexity of these associations, with age showing significant correlations in specific domains—particularly cognitive function, fatigue, pain perception, visual impairment, and bowel control—while other areas such as mental health, social support, and overall quality of life remained largely unaffected by age. Sexual satisfaction showed a weak but statistically significant positive association with age, suggesting that older adults may report stable or slightly improved satisfaction levels, potentially due to shifting expectations or greater emotional intimacy.

### 6.1. Fatigue and Age

Moderate positive correlations between age and scores on the MFIS were observed, indicating that older patients experience higher levels of fatigue. This is in accordance with studies attributing age-related fatigue to reduced physical reserves, comorbid conditions, and slower recovery following dialysis sessions [41,42].

### 6.2. Pain and Age

A moderate positive correlation between age and PES scores indicates that older individuals tend to report higher pain intensity. This is supported by prior research attributing increased pain in older dialysis patients to musculoskeletal and neuropathic conditions, as well as accumulated treatment burden [45,46].

### 6.3. Sexual Satisfaction and Age

A weak but statistically significant positive correlation was found between age and scores on the SSS, suggesting that older adults may report stable or even improved satisfaction despite reduced sexual activity. These results may reflect shifting expectations, greater emotional intimacy, or redefined priorities in older age [47,48,57].

### 6.4. Bowel Control and Age

A weak-to-moderate positive correlation was observed between age and BWCS scores, suggesting that older patients may face increased bowel function challenges. However, prior studies highlight that gastrointestinal symptoms in dialysis patients are multifactorial and may depend more on medication, dietary factors, and biochemical imbalances than on age alone [50,51].

### 6.5. Visual Impairment and Age

A weak-to-moderate positive correlation between age and IVIS scores was found, confirming that older dialysis patients report more vision-related difficulties. This is consistent with previous studies linking chronic kidney disease and aging to a higher incidence of conditions such as diabetic retinopathy and age-related macular degeneration [13,52,58].

### 6.6. Cognition and Age

Moderate positive correlations were found between age and all subscales of the PDQ, indicating that older individuals report greater cognitive difficulties, especially in attention, memory, and planning. These findings support earlier research identifying increased prevalence of cognitive impairment in older hemodialysis patients due to cerebrovascular changes and prolonged dialysis exposure [15,19,21].

### 6.7. Mental Health and Age

No significant relationship was observed between age and mental health outcomes as measured by the MHI, including its subscales and the abbreviated MHI-5. These results are consistent with prior findings suggesting that psychological well-being in hemodialysis patients is more strongly influenced by factors such as disease duration, treatment burden, social support, and coping mechanisms than by age itself [5,13,53].

### 6.8. Social Support and Age

The MSSS demonstrated weak, non-significant negative correlations with age, suggesting that perceived social support remains stable across different age groups. These findings align with literature emphasizing the protective role of social support regardless of age, although the sources and nature of support may vary between younger and older patients [22,25,54].

### 6.9. Quality of Life and Age

No statistically significant correlations were found between age and WHOQOL-BREF subscales, suggesting that age does not independently determine quality of life. While some studies report reduced physical functioning in older patients, others emphasize the role of psychological adaptation and external support systems in maintaining stable quality-of-life perceptions [2,53].

## 7. Conclusions

Our findings suggest that while age is an important variable in some clinical and psychosocial domains, it is not a universal determinant of patient outcomes in hemodialysis. Notably:

Cognitive impairment, fatigue, pain, and visual impairment increase with age, indicating a need for enhanced monitoring and supportive interventions for older patients.

Mental health, social support, and quality of life appear less affected by age, implying the relevance of individualized care strategies that account for psychological resilience and social dynamics.

The strong correlations between full and abbreviated versions of psychometric tools (MHI and MHI-5, PDQ and PDQ-5, MSSS and MSSS-5, and MFIS and MFIS-5) affirm their clinical value not only for professionals but also for use in patient education and self-assessment contexts. These short-form tools can be used during consultations to quickly assess well-being, and they can also serve as accessible instruments for patients to monitor their own symptoms over time, potentially improving engagement and adherence.

Further research should adopt longitudinal designs to explore causal pathways and track changes over time. Age-specific interventions addressing cognitive decline, fatigue, and pain management could improve patient functioning and quality of life.

In conclusion, age is associated with an increased burden in selected health domains among hemodialysis patients—particularly in cognitive, sensory, and fatigue-related parameters—while its impact on psychological well-being, social support, and life satisfaction is less pronounced. These findings underscore the importance of a multidimensional and individualized approach to dialysis care, ensuring that clinical strategies are responsive not only to biological aging but also to the broader psychosocial context of each patient.

## Figures and Tables

**Table 1:** Demographic, clinical, and dialysis-related characteristics of the study population.

		n/M [Range/%] (SD)
sex	male	47 [59.49%]
	female	32 [40.51%]
age		63.1 y [28–84] (12.2)
vascular access for hemodialysis	arteriovenous fistula	69 [87.3%]
	central venous catheter	10 [12.7%]
duration of hemodialysis		48.1 mths [1–317] (62.3)
marital status	married	55 [69.6%]
	single	24 [30.4%]
education	primary	45 [57.0%]
	secondary	21 [26.6%]
	university	13 [16.5%]
comorbidities	hypertension	53 [67.1%]
	diabetes	22 [27.8%]
kidney transplant in the past		6 [7.6%]
Kt/V		1.27 [0.34–2.50] (0.31)
ultrafiltration		2168 mL [400–4000] (950)
urea reduction ratio		0.65 [0.22–0.89] (0.09)

n—number; M—mean; SD—standard deviation.

**Table 2:** Correlation between age and MFIS scores.

Scale/Subscale	Pearson’s Correlation (r)	*p*-Value	Interpretation
Physical	0.514	<0.001	Moderate positive correlation, significant
Cognitive	0.531	<0.001	Moderate positive correlation, significant
Psychosocial	0.440	<0.001	Moderate positive correlation, significant
MFIS	0.523	<0.001	Moderate positive correlation, significant
MFIS-5	0.497	<0.001	Moderate positive correlation, significant

MFIS—Modified Fatigue Impact Scale, MFIS-5—abbreviated version of MFIS.

**Table 3:** Correlation between age and PES scores.

Scale	Pearson’s Correlation (r)	*p*-Value	Interpretation
PES	0.413	<0.001	Moderate positive correlation, significant, significant

PES—Pain Effects Scale.

**Table 4:** Correlation between age and SSS scores.

Scale	Spearman’s Correlation (ρ)	*p*-Value	Interpretation
SSS	0.284	0.017	Weak positive correlation, significant

SSS—Sexual Satisfaction Scale.

**Table 5:** Correlation between age and BWCS scores.

Scale	Spearman’s Correlation (ρ)	*p*-Value	Interpretation
BWCS	0.315	0.008	Moderate positive correlation, significant

BWCS—Bowel Control Scale.

**Table 6:** Correlation between age and IVIS scores.

Scale	Spearman’s Correlation (ρ)	*p*-Value	Interpretation
IVIS	0.370	0.002	Moderate positive correlation, significant

IVIS—Impact of Visual Impairment Scale.

**Table 7:** Correlation between age and PDQ scores.

Scale/Subscale	Spearman’s Correlation (ρ)	*p*-Value	Interpretation
Attention	0.421	<0.001	Moderate positive correlation, significant
Retrospective Memory	0.443	<0.001	Moderate positive correlation, significant
Prospective Memory	0.374	<0.001	Moderate positive correlation, significant
Planning	0.414	<0.001	Moderate positive correlation, significant
PDQ	0.445	<0.001	Moderate positive correlation, significant
PDQ-5	0.416	<0.001	Moderate positive correlation, significant

PDQ—Perceived Deficits Questionnaire, PDQ-5—abbreviated version of PDQ.

**Table 8:** Correlation between age and MHI scores.

Scale/Subscale	Spearman’s Correlation (ρ)	*p*-Value	Interpretation
Anxiety	0.098	0.240	Weak positive correlation, not significant
Depression	0.106	0.200	Weak positive correlation, not significant
Behavior Control	0.135	0.101	Weak positive correlation, not significant
Positive Affect	0.119	0.151	Weak positive correlation, not significant
MHI	0.122	0.139	Weak positive correlation, not significant
MHI-5	0.121	0.144	Weak positive correlation, not significant

MHI—Mental Health Inventory, MHI-5—abbreviated version of MHI.

**Table 9:** Correlation between age and MSSS scores.

Scale/Subscale	Spearman’s Correlation (ρ)	*p*-Value	Interpretation
Tangible Support	−0.078	0.435	Weak negative correlation, not significant
Emotional Support	−0.052	0.612	Weak negative correlation, not significant
Affectionate Support	−0.078	0.436	Weak negative correlation, not significant
Positive Social Interactions	−0.086	0.392	Weak negative correlation, not significant
MSSS	−0.065	0.525	Weak negative correlation, not significant
MSSS-5	−0.071	0.489	Weak negative correlation, not significant

MSSS—Modified Social Support Survey, MSSS-5—abbreviated version of MSSS.

**Table 10:** Correlation between age and WHOQOL-BREF scores.

Domains	Spearman’s Correlation (ρ)	*p*-Value	Interpretation
Physical	−0.103	0.247	Weak negative correlation, not significant
Psychological	0.037	0.717	Very weak positive correlation, not significant
Social Relationships	−0.046	0.646	Very weak negative correlation, not significant
Environment	−0.055	0.575	Very weak negative correlation, not significant

WHOQOL-BREF—World Health Organization Quality of Life questionnaire.

## Data Availability

The data presented in this study are available on request from the corresponding author. The data are not publicly available due to privacy restrictions.

## References

[1] Brown E.A., Zhao J., McCullough K., Fuller D.S., Figueiredo A.E., Bieber B., Finkelstein F.O., Shen J., Kanjanabuch T., Kawanishi H. (2021). Burden of Kidney Disease, Health-Related Quality of Life, and Employment Among Patients Receiving Peritoneal Dialysis and In-Center Hemodialysis: Findings from the DOPPS Program. Am. J. Kidney Dis..

[2] Chuasuwan A., Pooripussarakul S., Thakkinstian A., Ingsathit A., Pattanaprateep O. (2020). Comparisons of Quality of Life between Patients Underwent Peritoneal Dialysis and Hemodialysis: A Systematic Review and Meta-Analysis. Health Qual. Life Outcomes.

[3] Cogley C., Bramham J., Bramham K., Smith A., Holian J., O’Riordan A., Teh J.W., Conlon P., Mac Hale S., D’Alton P. (2023). High rates of psychological distress, mental health diagnoses and suicide attempts in people with chronic kidney disease in Ireland. Nephrol Dial Transpl..

[4] Kalender B., Ozdemir A.C., Dervisoglu E., Ozdemir O. (2007). Quality of life in chronic kidney disease: Effects of treatment modality, depression, malnutrition and inflammation. Int. J. Clin. Pract..

[5] Bhasin A.A., Molnar A.O., McArthur E., Nash D.M., Busse J.W., Cooper R., Heale E., Ip J., Pang J., Blake P.G. (2024). Mental Health and Addiction Service Utilization among People living with Chronic Kidney Disease. Nephrol. Dial. Transpl..

[6] Saedi F., Dehghan M., Mohammadrafie N., Xu X., Hermis A.H., Zakeri M.A. (2024). Predictive Role of Spiritual Health, Resilience, and Mental Well-Being in Treatment Adherence among Hemodialysis Patients. BMC Nephrol..

[7] Jorm A.F., Windsor T.D., Dear K.B., Anstey K.J., Christensen H., Rodgers B. (2005). Age Group Differences in Psychological Distress: The Role of Psychosocial Risk Factors That Vary with Age. Psychol. Med..

[8] Ginieri-Coccossis M., Theofilou P., Synodinou C., Tomaras V., Soldatos C. (2008). Quality of Life, Mental Health and Health Beliefs in Haemodialysis and Peritoneal Dialysis Patients: Investigating Differences in Early and Later Years of Current Treatment. BMC Nephrol..

[9] Sułkowski L., Matyja A., Matyja M. (2025). The Impact of Dialysis Duration on Multidimensional Health Outcomes: A Cross-Sectional Study. J. Clin. Med..

[10] Palmer S.C., Vecchio M., Craig J.C., Tonelli M., Johnson D.W., Nicolucci A., Pellegrini F., Saglimbene V., Logroscino G., Hedayati S.S. (2013). Association between Depression and Death in People with CKD: A Meta-Analysis of Cohort Studies. Am. J. Kidney Dis..

[11] Smith D.J., Martin D., McLean G., Langan J., Guthrie B., Mercer S.W. (2013). Multimorbidity in Bipolar Disorder and Undertreatment of Cardiovascular Disease: A Cross-Sectional Study. BMC Med..

[12] Golenia A., Olejnik P., Żołek N., Wojtaszek E., Małyszko J. (2023). Cognitive Impairment and Anxiety Are Prevalent in Kidney Transplant Recipients. Kidney Blood Press. Res..

[13] Sulkowski L., Rubinkiewicz M., Matyja A., Matyja M. (2023). Visual Impairment in Hemodialyzed Patients—An IVIS Study. Medicina.

[14] O’Lone E., Connors M., Masson P., Wu S., Kelly P.J., Gillespie D., Parker D., Whiteley W., Strippoli G.F., Palmer S.C. (2016). Cognition in People with End-Stage Kidney Disease Treated with Hemodialysis: A Systematic Review and Meta-Analysis. Am. J. Kidney Dis..

[15] Zhang J., Wu L., Wang P., Pan Y., Dong X., Jia L., Zhang A. (2024). Prevalence of Cognitive Impairment and Its Predictors among Chronic Kidney Disease Patients: A Systematic Review and Meta-Analysis. PLoS ONE.

[16] Drew D.A., Weiner D.E., Sarnak M.J. (2019). Cognitive Impairment in CKD: Pathophysiology, Management, and Prevention. Am. J. Kidney Dis..

[17] Wang Y., Qiu Y., Ren L., Jiang H., Chen M., Dong C. (2024). Social Support, Family Resilience and Psychological Resilience among Maintenance Hemodialysis Patients: A Longitudinal Study. BMC Psychiatry.

[18] Polinder-Bos H.A., Garcia D.V., Kuipers J., Elting J.W.J., Aries M.J.H., Krijnen W.P., Groen H., Willemsen A.T.M., van Laar P.J., Strijkert F. (2018). Hemodialysis Induces an Acute Decline in Cerebral Blood Flow in Elderly Patients. J. Am. Soc. Nephrol..

[19] Findlay M.D., Dawson J., Dickie D.A., Forbes K.P., McGlynn D., Quinn T., Mark P.B. (2019). Investigating the Relationship between Cerebral Blood Flow and Cognitive Function in Hemodialysis Patients. J. Am. Soc. Nephrol..

[20] Ziesat H.A., Logue P.E., McCarty S.M. (1980). Psychological Measurement of Memory Deficits in Dialysis Patients. Percept. Mot. Ski..

[21] Drew D.A., Weiner D.E., Tighiouart H., Scott T., Lou K., Kantor A., Fan L., Strom J.A., Singh A.K., Sarnak M.J. (2015). Cognitive Function and All-Cause Mortality in Maintenance Hemodialysis Patients. Am. J. Kidney Dis..

[22] Abdel-Kader K., Unruh M.L., Weisbord S.D. (2009). Symptom Burden, Depression, and Quality of Life in Chronic and End-Stage Kidney Disease. Clin. J. Am. Soc. Nephrol..

[23] Fletcher B.R., Damery S., Aiyegbusi O.L., Anderson N., Calvert M., Cockwell P., Ferguson J., Horton M., Paap M.C.S., Sidey-Gibbons C. (2022). Symptom Burden and Health-Related Quality of Life in Chronic Kidney Disease: A Global Systematic Review and Meta-Analysis. PLoS Med..

[24] Kang G.W., Lee I.H., Ahn K.S., Lee J., Ji Y., Woo J. (2015). Clinical and Psychosocial Factors Predicting Health-Related Quality of Life in Hemodialysis Patients. Hemodial. Int..

[25] Sułkowski L., Matyja A., Matyja M. (2024). Social Support and Quality of Life in Hemodialysis Patients: A Comparative Study with Healthy Controls. Medicina.

[26] Fisk J.D., Ritvo P.G., Ross L., Haase D.A., Marrie T.J., Schlech W.F. (1994). Measuring the Functional Impact of Fatigue: Initial Validation of the Fatigue Impact Scale. Clin. Infect. Dis..

[27] Ritvo P.G., Fischer J.S., Miller D.M., Andrews H., Paty D.W., LaRocca N.G. (1997). MSQLI Multiple Sclerosis Quality of Life Inventory: A User’s Manual.

[28] Fisk J.D., Pontefract A., Ritvo P.G., Archibald C.J., Murray T.J. (1994). The Impact of Fatigue on Patients with Multiple Sclerosis. Can. J. Neurol. Sci..

[29] Moulin D.E., Foley K.M., Ebers G.C. (1988). Pain Syndromes in Multiple Sclerosis. Neurology.

[30] Archibald C.J., McGrath P., Ritvo P.G., Fisk J.D., Bhan V., Maxner C.E., Murray T.J. (1994). Pain prevalence, severity and impact in a clinic sample of multiple sclerosis patients. Pain.

[31] Nowinski J.K., LoPiccolo J. (1979). Assessing Sexual Behavior in Couples. J. Sex Marital Ther..

[32] Chia Y.W., Fowler C.J., Kamm M.A., Henry M.M., Lemieux M.C., Swash M. (1993). Prevalence of bowel dysfunction in patients with multiple sclerosis and bladder dysfunction. J. Neurol..

[33] Sullivan J.J., Edgley K., Dehoux E. (1990). A Survey of Multiple Sclerosis: Part 1: Perceived Cognitive Problems and Compensatory Strategy Use. Can. J. Rehabil..

[34] Rao S.M., Leo G.J., Bernadin L., Unverzagt F. (1991). Cognitive Dysfunction in Multiple Sclerosis: I. Frequency, Patterns, and Prediction. Neurology.

[35] Veit C.T., Ware J.E. (1983). The Structure of Psychological Distress and Well-Being in General Populations. J. Consult. Clin. Psychol..

[36] Stewart A.L., Ware J.E. (1992). Measuring Functioning and Well-Being: The Medical Outcomes Study Approach.

[37] Sherbourne C.D., Stewart A.L. (1991). The MOS Social Support Survey. Soc. Sci. Med..

[38] The WHOQOL Group (1998). Development of the World Health Organization WHOQOL-BREF Quality of Life Assessment. Psychol. Med..

[39] Spearman C. (1987). The proof and measurement of association between two things. By C. Spearman, 1904. Am. J. Psychol..

[40] Pearson K. (1895). Notes on Regression and Inheritance in the Case of Two Parents. Proc. R. Soc. Lond..

[41] Al Naamani Z., Gormley K., Noble H., Santin O., Al Maqbali M. (2021). Fatigue, Anxiety, Depression and Sleep Quality in Patients Undergoing Haemodialysis. BMC Nephrol..

[42] Artom M., Moss-Morris R., Caskey F., Chilcot J. (2014). Fatigue in Advanced Kidney Disease. Kidney Int..

[43] Biniaz V., Tayybi A., Nemati E., Sadeghi Shermeh M., Ebadi A. (2013). Different Aspects of Fatigue Experienced by Patients Receiving Maintenance Dialysis in Hemodialysis Units. Nephrourol. Mon..

[44] Maruyama Y., Nakayama M., Ueda A., Miyazaki M., Yokoo T. (2021). Comparisons of Fatigue Between Dialysis Modalities: A Cross-Sectional Study. PLoS ONE.

[45] Brkovic T., Burilovic E., Puljak L. (2018). Risk Factors Associated with Pain on Chronic Intermittent Hemodialysis: A Systematic Review. Pain Pract..

[46] Fleishman T.T., Dreiher J., Shvartzman P. (2018). Pain in Maintenance Hemodialysis Patients: A Multicenter Study. J. Pain Symptom Manag..

[47] Iacovides A., Fountoulakis K.N., Balaskas E., Manika A., Markopoulou M., Kaprinis G., Tourkantonis A. (2002). Relationship of Age and Psychosocial Factors with Biological Ratings in Patients with End-Stage Renal Disease Undergoing Dialysis. Aging Clin. Exp. Res..

[48] Fugl-Meyer K.S., Nilsson M., Hylander B., Lehtihet M. (2017). Sexual Function and Testosterone Level in Men with Conservatively Treated Chronic Kidney Disease. Am. J. Mens Health.

[49] Mor M.K., Sevick M.A., Shields A.M., Green J.A., Palevsky P.M., Arnold R.M., Fine M.J., Weisbord S.D. (2014). Sexual Function, Activity, and Satisfaction Among Women Receiving Maintenance Hemodialysis. Clin. J. Am. Soc. Nephrol..

[50] Gök E.G., İnci A., Çoban M., Kutsal D.A., Kürşat S. (2017). Functional Bowel Disorders and Associated Risk Factors in Hemodialysis Patients in Turkey. Turk. J. Gastroenterol..

[51] Wu M.J., Chang C.S., Cheng C.H., Chen C.H., Lee W.C., Hsu Y.H., Shu K.H., Tang M.J. (2004). Colonic Transit Time in Long-Term Dialysis Patients. Am. J. Kidney Dis..

[52] Nusinovici S., Sabanayagam C., Teo B.W., Tan G.S., Wong T.Y. (2019). Vision Impairment in CKD Patients: Epidemiology, Mechanisms, Differential Diagnoses, and Prevention. Am. J. Kidney Dis..

[53] Untas A., Thumma J., Rascle N., Rayner H., Mapes D., Lopes A.A., Fukuhara S., Akizawa T., Morgenstern H., Robinson B.M. (2011). The Associations of Social Support and Other Psychosocial Factors with Mortality and Quality of Life in the Dialysis Outcomes and Practice Patterns Study. Clin. J. Am. Soc. Nephrol..

[54] McClellan W.M., Stanwyck D.J., Anson C.A. (1993). Social Support and Subsequent Mortality Among Patients with End-Stage Renal Disease. J. Am. Soc. Nephrol..

[55] Zimet G.D., Powell S.S., Farley G.K., Werkman S., Berkoff K.A. (1990). Psychometric Characteristics of the Multidimensional Scale of Perceived Social Support. J. Pers. Assess..

[56] Cella D., Riley W., Stone A., Rothrock N., Reeve B., Yount S., Amtmann D., Bode R., Buysse D., Choi S. (2010). The Patient-Reported Outcomes Measurement Information System (PROMIS) developed and tested its first wave of adult self-reported health outcome item banks: 2005-2008. J. Clin. Epidemiol..

[57] Al Eissa M., Al Sulaiman M., Jondeby M., Karkar A., Barahmein M., Shaheen F.A., Al Sayyari A. (2010). Factors Affecting Hemodialysis Patients’ Satisfaction with Their Dialysis Therapy. Int. J. Nephrol..

[58] Müller M., Schönfeld C.L., Grammer T., Krane V., Drechsler C., Genser B., Kohnen T., Wanner C., März W. (2020). Risk Factors for Retinopathy in Hemodialysis Patients with Type 2 Diabetes Mellitus. Sci. Rep..

